# A guideline for 3D printing terminology in biomedical research utilizing ISO/ASTM standards

**DOI:** 10.1186/s41205-021-00098-5

**Published:** 2021-03-22

**Authors:** Amy E. Alexander, Nicole Wake, Leonid Chepelev, Philipp Brantner, Justin Ryan, Kenneth C. Wang

**Affiliations:** 1 Department of Radiology, Mayo Clinic, Rochester, MN USA; 2 Department of Radiology, Montefiore Medical Center, Albert Einstein College of Medicine, 111 East 210th Street, Bronx, NY 10467 USA; 3 Department of Radiology, NYU Langone Health, NYU Grossman School of Medicine, Center for Advanced Imaging Innovation and Research (CAI2R) and Bernard and Irene Schwartz Center for Biomedical Imaging, New York, NY USA; 4 Department of Radiology, Stanford University, Stanford, CA USA; 5 Department of Radiology, University Hospital Basel, Basel, Switzerland; 6 3D Innovations Lab, Rady Children’s Hospital, San Diego, CA USA; 7 Department of Diagnostic Radiology and Nuclear Medicine, University of Maryland School of Medicine, Baltimore, MD USA; 8 Imaging Service, Baltimore VA Medical Center, Baltimore, MD USA

## Abstract

First patented in 1986, three-dimensional (3D) printing, also known as additive manufacturing or rapid prototyping, now encompasses a variety of distinct technology types where material is deposited, joined, or solidified layer by layer to create a physical object from a digital file. As 3D printing technologies continue to evolve, and as more manuscripts describing these technologies are published in the medical literature, it is imperative that standardized terminology for 3D printing is utilized. The purpose of this manuscript is to provide recommendations for standardized lexicons for 3D printing technologies described in the medical literature. For all 3D printing methods, standard general ISO/ASTM terms for 3D printing should be utilized. Additional, non-standard terms should be included to facilitate communication and reproducibility when the ISO/ASTM terms are insufficient in describing expository details. By aligning to these guidelines, the use of uniform terms for 3D printing and the associated technologies will lead to improved clarity and reproducibility of published work which will ultimately increase the impact of publications, facilitate quality improvement, and promote the dissemination and adoption of 3D printing in the medical community.

## Introduction

Three-dimensional (3D) printing, also known as additive manufacturing or rapid prototyping, refers to a process of creating a physical object from a 3D digital model, typically by laying down or solidifying a material layer by layer in succession. First reported in 1986 [1], 3D printing now comprises many distinct printing technologies used in a wide range of industries. With this growth has come a proliferation of terms used to refer to 3D printing technologies, where some of these terms are commercial trademarks, non-standard terms, and/or poorly defined terms.

The need for standardized terminology has long been recognized in clinical research and medical practice, because a common vocabulary promotes clarity and reproducibility. Within imaging, standard lexicons have been developed for a variety of imaging technologies [2–4] as well as for the clinical interpretation of imaging exams [5–8].

Standard terminology in the domain of clinical 3D printing has been an important goal since the founding of the journal 3D Printing in Medicine [9], and a comprehensive analysis of the literature led to the recommendation that the term “3D printing” be adopted as an inclusive term covering technologies also described with other terms such as “rapid prototyping” and “additive manufacturing” [10]. Recent work by the Radiological Society of North America (RSNA) has brought 3D printing terms to the RadLex project, a radiological ontology for use in reporting, decision support, data mining, education, and research [11].

As 3D printing technologies continue to evolve, the importance of standard terminology increases. An analogous situation may be found in the development of magnetic resonance imaging (MRI). As MRI became more commonplace and the term “nuclear” was dropped from the lexicon, leaders and professional societies recognized the need to establish a standard technical language for these techniques [12, 13]. Similarly, promoting a standard lexicon in the domain of 3D printing will serve to enhance work in this area in many ways. Use of uniform terms for indexing of the literature will lead to improved discoverability of related work, and also improved clarity in the exchange of ideas, techniques and results. The most immediate need is for standardized terms to refer to the different types of 3D printing technologies.

### Current state

As clinicians, researchers, and scientists around the world publish their work in clinical 3D printing, it is clear that the usage of terms varies widely. Some authors utilize common trademarked terms for describing a print technology (e.g., fused deposition modeling, or “FDM”, is a trademark of Stratasys, not a general technology descriptor) [14]. Other authors use less-common synonyms for key terms (e.g., “rapid prototyping” instead of “3D printing”). The journal 3D Printing in Medicine and its articles can be made more consistent through the adoption of standard nomenclature.

There are existing standards for 3D printing terminology. The foremost standard is the “ISO/ASTM 52900 Standard Terminology for Additive Manufacturing – General Principles – Terminology”, which replaced “ASTM 52900:2015 Standard Terminology for Additive Manufacturing Technologies” in March of 2020 [15]. With regard to 3D printing technologies, this standard defines seven distinct types of 3D printing processes. Courses like MIT’s Additive Manufacturing for Innovative Design and Production have adopted this nomenclature [16]. In addition, accrediting bodies such as SME (formerly the Society of Manufacturing Engineers) rely on this nomenclature to certify individuals to work competently in this space through the SME Additive Manufacturing Certification [17]. Adhering to such standards is a natural way to extend the consistency of the published literature.

Table 1 lists the seven standard terms for 3D printing technology types described by ISO/ASTM 52900 along with several additional fields. The first column shows the ISO/ASTM term itself. For each such term, one or more additional terms related to the standard term are shown in the second column. These additional terms include commercial examples of the given technology type, and non-standard synonyms or subtypes of the technology type. A brief description of each standard term is provided by the authors in the third column for quick reference. However, readers are encouraged to review ISO/ASTM 52900 for formal definitions. The final column lists the associated RadLex identifier for each standard term.

**Table 1:** Generalized standard terms for 3D printing technologies. For each standard term, one or more commercial and other terms are listed, along with a description of the technology type, and the associated identifier in the RadLex terminology. Please refer to the ISO/ASTM standard for a complete description of each generalized term [15]

Generalized Standard Term	Commercial and Other Term Examples	Description	RadLex Identifier
Binder Jetting	• ProJet Color Jet Printing (CJP)	Liquid agents are selectively dropped onto powder media. Subsequent infiltration or heating may be required.	RID50562
Directed Energy Deposition	• Laser Engineered Net Shape (LENS) • Electron Beam Additive Manufacture (EBAM)	Focused application of energy and material selectively melted and fused on a build platform or part.	RID50563
Material Extrusion	• Fused Deposition Modeling (FDM) • Fused Filament Fabrication (FFF)	Material is dispensed, often through a heated nozzle, onto a build platform.	RID50564
Material Jetting	• Nano particle Jetting (NPJ) • Drop-On-Demand (DOD) • PolyJet • ProJet Multijet Printing (MJP)	A print head dispenses droplets of media, usually a photopolymer, onto a build platform where each layer is solidified or cured.	RID50565
Powder Bed Fusion	• Selective Laser Sintering (SLS) • Selective Laser Melting (SLM) • Direct Metal Printing (DMP) • Direct Metal Laser Sintering (DMLS) • Electron Beam Melting (EBM) • Multi Jet Fusion (MJF)	Powder media is deposited on a build platform and subsequently bonded together through a heating process.	RID50566
Sheet Lamination	• Laminated Object Manufacturing (LOM)	Discrete layers of material are fused or glued together to form an object.	RID50567
Vat Photopolymerization	• Stereolithography apparatus (SLA) • Direct Light Processing (DLP) • Continuous liquid interface production (CLIP)	Liquid photopolymer is selectively exposed to a light source facilitating layer-by-layer curing.	RID50568

### Evaluation

In order to evaluate terminology usage, a review of the 61 articles published in 3D Printing in Medicine, Springer Nature, from its inception through May 2020 was performed [18]. First, titles were assessed for the presence of the term “3D printing” and any synonyms. The published papers were also reviewed to determine if “3D printing” and ISO/ASTM generalized standard terms were used in the text of the manuscript; if not, any alternative terms utilized were noted. 49 (80.3% of all published manuscripts) of these referenced “3D printing” in the title; and 58 manuscripts (95.1%) mentioned “3D printing” in the body of the manuscript at least once. Other common synonyms included “additive manufacturing,” “modeling,” and “rapid prototyping.” With regard to 3D printing technologies, these papers commonly refer to specific machines without identifying a technology type. These papers also commonly use trademarked terms such as “polyjet,” “stereolithography,” and “fused deposition modeling.” In some cases, descriptions of material properties (e.g. “multicolor”) are presented as a proxy for the printing technology used. Only 6 (9.8%) manuscripts reference specific 3D printing technologies using ISO/ASTM standardized nomenclature. The inconsistent use of terminology illustrated here creates an impediment for researchers.

## Recommendations

In order to promote consistency in the literature, the term “3D printing” should be used in manuscripts instead of synonyms such as “additive manufacturing” or “rapid prototyping.” Similarly, authors should take note of the standard general ISO/ASTM terms for 3D printing technologies and apply these as appropriate. Any research communication or statement pertaining to the set of technologies encompassed by a given standard ISO/ASTM term should always utilize the appropriate standard term. For example, a review broadly discussing vat photopolymerization technologies should use the standard term “vat photopolymerization” rather than terms that may either denote a specific implementation of a more general technology or act as incomplete synonyms due to an inappropriately narrow reference to a set of specific patented technologies, such as stereolithography (SLA), continuous liquid interface production (CLIP), or direct light processing (DLP). In this setting, the use of non-standard terms hinders research discoverability and inappropriately constricts the scope of the communication. In all cases, terms identifying specific 3D printing technology implementations should be accompanied by the corresponding general standard ISO/ASTM technology type with its first mention and where appropriate throughout the manuscript. For example, consider a manuscript evaluating the relative accuracy of printers utilizing the CLIP subtype of the broader vat photopolymerization technology type. It would be inappropriate for such a manuscript to assert that observations derived using CLIP printers are valid for all vat photopolymerization printers, and the more specific term to include the trade name of the 3D printer should be used so that the readership will know the name of the printer used. However, it would still be important for authors to note that CLIP is a subtype of the standard technology type vat photopolymerization.

Where the intent of a research communication is to refer to a specific set of printing technologies, non-standard commercial or non-commercial terms should be used to facilitate communication and reproducibility. The purpose is to be descriptive and consistent, as opposed to supporting or marketing any particular product. 3D printing is inherently different than the “MRI” example alluded to above because technologies (hardware, software, materials) use different physics to create the 3D printed part. If an anatomic model or an anatomic guide is printed on a Form3 printer, the generalized standard term is vat photopolymerization and the Form3 printer should be noted. For example, a model of the left atrial appendage was printed using vat photopolymerization (Form3, Formlabs, Cambridge, MA). Table 2 describes additional examples of 3D printer referencing. The Radiological Society of North America-American College of Radiology (RSNA-ACR) 3D printing registry includes a data dictionary and the registry recognizes via drop-down menus 108 3D printers across 13 manufacturers with their respective generalized standard terms [19, 20].

**Table 2:** Suggested prose for 3D Printing in Medicine: “A 3D printed model was printed with insert technology type (insert commercial name, insert manufacturer name, insert manufacturer headquarters).” Note that a Directed Energy Deposition (DED) example was not included as there is not currently a DED printer in the RSNA-ACR 3D printing registry data dictionary

Generalized Standard Term	Example Printer	Manufacturer	Prose
Binder Jetting	ProJet CJP 660 Pro	3D Systems	A 3D printed model was printed with binder jetting (ProJet CJP 660 Pro, 3D Systems, Rock Hill, SC).
Material Extrusion	S5	Ultimaker	A 3D printed model was printed with material extrusion (S5, Ultimaker, Utrecht, Netherlands)
Material Jetting	J750	Stratasys	A 3D printed model was printed with material jetting (J750, Stratasys, Eden Prairie, MN).
Powder Bed Fusion	Jet Fusion 580	HP	A 3D printed model was printed with powder bed fusion (Jet Fusion 580, HP, Palo Alto, CA)
Sheet Lamination	SLCOM 1	EnvisionTEC	A 3D printed model was printed with sheet lamination (SLCOM1, EnvisionTEC, Gladbeck, Germany).
Vat Photopolymerization	Form3	Formlabs	A 3D printed model was printed with vat photopolymerization (Form 3, Formlabs, Cambridge, MA)

In accordance with widely established publication practices, the use of commercial trademarks in publication titles and manuscript text is permitted to facilitate communication. Numerous examples of such utilization of commercial trademarks exist. For example, within medical imaging literature, numerous publications incorporate names of trademarked MRI sequences within titles and article text [21–25]. However, the use of any subjective and/or scientifically unsubstantiated positive or negative language in relation to commercial or trademarked product descriptors is unacceptable; reviewers and editors should recognize such language and require that manuscripts be edited accordingly. Authors should avoid subjective adjectives that are not supported by data and make changes when requested. For example, a commercial technology or product should not be described as ‘user-friendly’, ‘accurate’, or ‘reliable’ outside of the context of objective assessments establishing such descriptors.

### Titles and content

Manuscripts should use the term “3D printing” in the title and in the main body of the text, instead of synonyms such as “additive manufacturing,” “rapid prototyping,” or “3D manufacturing.” This use represents a divergence from the ISO/ASTM standard which uses the term “additive manufacturing”. “3D printing” has far exceeded other terms with regard to adoption and use by the global medical and non-medical community. This descriptivist approach to promoting “3D printing” above other terms will enhance indexing and searching capabilities of readers.

As detailed above, manuscripts using non-standard terms and trademarks (e.g., “fused deposition modeling”, “polyjet”, “multi jet fusion”) should use the ISO/ASTM term instead (e.g., “material extrusion”, “material jetting”, “powder bed fusion” respectively) in the title as needed and throughout the manuscript. In cases where reference to a more specific commercial term or other non-standard term constitutes an important element of the work presented, such terms should be used in conjunction with the relevant ISO/ASTM term (Table 3).

**Table 3:** Examples of title and content corrections to comply with the recommendations herein

	Example	Problem	Recommendation
Title A:	Mechanical Load on Sterilized Rapid Prototyped Prosthetic Valve	Readers searching for “3D Printing & Valve” would fail to see this publication.	Mechanical Load on Sterilized 3D Printed Prosthetic Valve
Content A:	Polyjet was used to generate a model of the valve.	Readers may not be familiar with Polyjet as a term associated with material jetting.	A material jetting printer, (Objet500 Connex 3, Stratasys, Eden Prairie, MN) was used to generate a model of the valve.
Title B:	Simulated Surgical Osteotomies on 3D Powder Maxillary Models	Insufficient specificity regarding type of printing technology. Readers may interpret “powder” as referring to binder jetting, or powder bed fusion.	Simulated Surgical Osteotomies on 3D Printed Maxillary Models Created with Binder Jetting
Content B:	A ProJet 660Pro produced a “sandstone” replica of the zygomatic arch.	Referencing printer name only does not indicate the technology type.	A binder jetting printer (ProJet 660Pro, 3D Systems, Rock Hill, SC) produced a “sandstone” (gypsum-based) replica of the zygomatic arch.
Title C:	Additive Manufacturing of Liver Models for Education	Use of “3D Printing” in lieu of “Additive Manufacturing” is recommended.	3D Printing of Liver Models for Education
Content C:	SLA was used to 3D print clear liver models.	Readers may not know that SLA is stereolithography apparatus, a term that falls under the vat photopolymerization technology type.	A vat photopolymerization printer (NP1, NewPro3D, Vancouver, British Columbia, Canada), using RG35 resin, was used to 3D print clear liver models.

Authors are recommended to specifically cite the technologies, the printers (make, model), and types of printing materials (feedstock) and other post-processing consumables utilized in the research reported. However, given the rapid and on-going evolution of printers, feedstock materials, and consumables, authors should expect that readers may be unfamiliar with the specific technologies, brands and machines mentioned in a given manuscript. It is suggested that authors include the generalized ISO/ASTM nomenclature at least once per technology reported in a manuscript.

Descriptions of material properties should not be used as proxies for 3D printing technologies. Rather, materials and technologies should be described separately.

## Conclusions

By following these recommendations, authors working in medical 3D printing will improve the clarity and reproducibility of their work. The expanding literature in this field will be easier to search. Researchers and clinical users will have enhanced capabilities to interpret published results. These benefits will improve the value of publications, facilitate quality improvement, and promote the dissemination and adoption of 3D printing in the medical community.
